# Leveraging technology in public-private partnerships: a model to address public health inequities

**DOI:** 10.3389/frhs.2023.1187306

**Published:** 2023-06-13

**Authors:** Angel Arnaout, Melina Oseguera-Arasmou, Nikesh Mishra, Bennett M. Liu, Ahanjit Bhattacharya, David C. Rhew

**Affiliations:** ^1^ School of Medicine, Stanford University, Stanford, CA, United States; ^2^ Department of Chemistry, Stanford University, Stanford, CA, United States; ^3^ Healthcare, Microsoft Corporation, Redmond, WA, United States; ^4^ Division of Primary Care and Population Health, Department of Medicine, Stanford University, Stanford, CA, United States

**Keywords:** public-private partnership, health inequity, technology, community based organizations, value creation

## Abstract

Long-standing inequities in healthcare access and outcomes exist for underserved populations. Public-private partnerships (PPPs) are where the government and a private entity jointly invest in the provision of public services. Using examples from the Health Equity Consortium (HEC), we describe how technology was used to facilitate collaborations between public and private entities to address health misinformation, reduce vaccine hesitancy, and increase access to primary care services across various underserved communities during the COVID-19 pandemic. We call out four enablers of effective collaboration within the HEC-led PPP model, including: 1. Establishing trust in the population to be served 2. Enabling bidirectional flow of data and information 3. Mutual value creation and 4. Applying analytics and AI to help solve complex problems. Continued evaluation and improvements to the HEC-led PPP model are needed to address post-COVID-19 sustainability.

## 1. Introduction

The COVID-19 pandemic has highlighted the persistent health inequities that permeate our health system: lack of access to routine care and affordable healthcare insurance, and worsened health outcomes for vulnerable, under-served and under-represented populations in the US (1). Of particular significance during the pandemic was the disproportionate burden of COVID-19 and its consequences in these groups— including higher rates of exposure, transmission, severity of illness, and mortality, accompanied by lower COVID-19 testing and vaccination rates(2).

Traditional initiatives to address health inequities in the US have included (1) raising awareness to the public through education about health equity (3) (2) improving resource provisions to populations most harmed by health disparities (4) and (3) offering cultural competency training to healthcare providers (5). Less commonly explored is the use of public–private partnerships (PPPs) to address health inequities (6), although the COVID-19 pandemic has demonstrated how important it is that the public and private sectors join together during an emergency response (7). PPPs are where the government and a private entity jointly invest in the provision of public services. Through this arrangement, the private sector takes on significant financial, technical and operational risks while the public entity is held accountable for defined outcomes (8). PPPs are common in nonhealthcare sectors of the economy (such as infrastructure, transportation and energy) and typically seek to capture private sector capital or expertise to improve provision of a public service. In healthcare, the PPP approach can be applied to a wide range of healthcare system needs including construction of facilities, provision of medical equipment or supplies, or delivery of healthcare services across the continuum of care. Of particular interest in this paper is the role of government agencies partnering with private technology companies to facilitate the translation of health data into actionable insights to streamline operations, improve care coordination, and enable greater insights.

The Health Equity Consortium (HEC) (9) is a program of the California Health Medical Reserve Corps (CHMRC) exploring innovative solutions to address the needs of vulnerable populations and public health. Using the PPP model, HEC has formalized connections between community-based organizations (CBOs), local healthcare, public health, healthcare payers, lifescience partners and partners in the technology space (9, 10). HEC's shared-risk, shared-cost model seeks to overcome structural care gaps and break delivery silos while building trust in the populations being served. Operating as an extension of public health and fully engaging healthcare, community-based organizations, and payers, their mission is focused around these four gap areas:

• **Convening Organizations**: Fostering community-level collaboration with PPPs addressing complex, ecosystem-level health equity challenges on multiple fronts.
• **Enabling Data Collaboration:** Providing secure, privacy preserving, trusted technology solutions for community-level health equity data collection, bi-directional sharing, replacing manual with electronic case reporting, analytics, geospatial observations, and surveillance dashboards.
• **Mobilizing Communities:** Building community-level capability to successfully execute and scale health equity initiatives appropriate for each community and aligned with regional initiatives for sustainable impact, improved outcomes while building stronger, more resilient communities.
• **Navigating Social Care:** Helping underserved populations navigate the complexities of fragmented social care programs with consistent messaging and information across multiple touch points.

**Figure 1:**
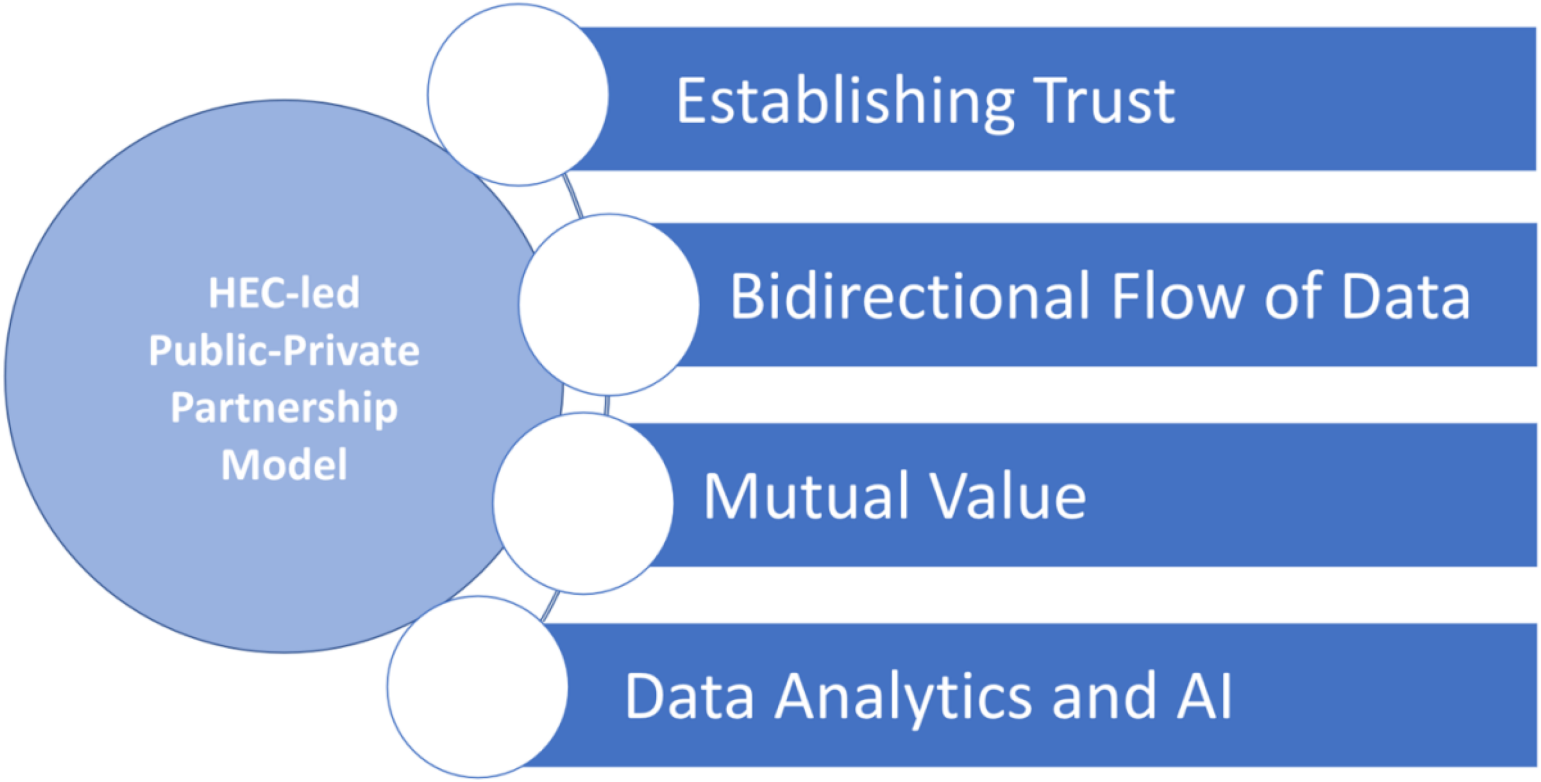
Key enablers of success of the public-private partnership model in addressing health inequity.

The HEC is now using this PPP model to expand beyond COVID vaccinations to address primary care challenges such as childhood immunizations, blood pressure, glucose monitoring, and cancer screenings in communities, including in Georgia and Mississippi. The common challenge faced by the public health departments is that health misinformation, hesitancy to care, and inequities in access resulted in health disparities in its vulnerable populations that public health departments could not solve alone. This paper expands on the key enablers of an effective collaboration within a PPP model that aims to address health inequities (Figure 1).

## 2. Key enablers of success of the PPP model in addressing health equity

### 2.1. Establishing trust in the population to be served

Health equity is a complex multi-sectoral issue which requires multiple stakeholders to address. Traditional efforts have mostly been focused on healthcare providers and the government (county and state public health agencies). For many under-represented communities, however, language barriers, low literacy and health literacy rates, concerns about deportation, cost (including lack of health insurance), lack of access to healthcare providers, and lack of computer and cell phone access are key barriers to achieving health equity. Addressing these challenges requires partnership with those who are attuned to the particular needs of the local population and have a long-standing trusted relationship with the community. CBOs, not-for-profit organizations, faith-based organizations, and non-government organizations who are empowered to engage community members through their established relationships can be trusted, knowledgeable partners. They provide the necessary input, guidance, and active support to engage members of the community

One example of an initiative where collaboration with trusted partners was important was when HEC partnered with Microsoft and local CBOs to improve COVID-19 testing and vaccinations rates in King county, Washington State. There were two pressing issues in this community in early 2021 (10, 11). The first was the need to significantly increase the number of vaccinations delivered to the public, in line with the increasing vaccine supply; and the second was to ensure that the minority and underserved populations who had worse outcomes from COVID-19 (such as the Latino and indigenous people of color (BIPOC), had access to vaccinations. HEC worked with Microsoft to develop a scheduling portal, two apps to track registration and schedule second doses, an automated appointment email system, and a syringe QR code printer and scanner—all at the same time. Using existing infrastructure of local CBOs, HEC brought COVID-19 testing and vaccinations to the local residents, meeting residents where they work or gather, including malls, farms, churches, and other community locations, such as farmer's markets and health hubs. HEC leveraged Microsoft's technology to scale up the volume of vaccinations performed that otherwise would not have been achieved by the CBO alone. Microsoft's QR code solutions allowed patients to move quickly through the site and did not require them to show ID at any point, making the experience more accessible for those with privacy concerns or documentation hurdles such as in the minority and vulnerable populations. The team also configured patient registration systems between two of the top medical centers in King County, Evergreen Health and Overlake Medical Center to seamlessly communicate with one another. Live Power BI dashboards helped medical professionals measure the right amount of vaccine to meet a given day's demand, preventing any waste or shortages. And the dashboards also helped those transporting the vaccine understand when and where to bring the mixtures. In partnership with community organizations such as Centro Cultural Mexicano, Microsoft supported a culturally appropriate pop-up vaccination events for those without reliable transportation or traditional methods of registering for appointments. Going into neighborhoods to build culturally sensitive communication campaigns, Microsoft helped HEC, CBOs and the coalition of healthcare partners achieve greater success in increasing vaccination rates than they otherwise would have achieved alone. Within this initiative, HEC, Microsoft and community stakeholders also assisted individuals and families to navigate local health and social care. From assessing eligibility and enrolling individuals in available social programs (e.g., Medicaid, CHIP, SNAP, WIC, TANF, others), to evaluating an individual's social needs. Connections with appropriate local social care programs and clinical care settings were facilitated.

Employing this collaborative, capacities-centric approach, healthcare and related services were brought to underserved communities where and how they were needed.

### 2.2. Real time, bidirectional flow and sharing of data

A major challenge faced by traditional collaborative partnerships that address health equity is the lack of infrastructure to collect and share data across health care and social services settings in an accurate and efficient manner. Data sharing, especially if done in real time, allows community stakeholders to learn from each other and collaborate on shared priorities. One of the key enablers for HEC's success in its projects was the technology that enabled bi-directional flow of data between CBOs, public health, and local healthcare organizations. The focus on public health and community-wide data complements prior successful work to share of clinical health information through health information exchange is funded through the American Recovery and Reinvestment Act of 2009 (ARRA) and the Health Information Technology for Economic and Clinical Health (HITECH) Act (12).

Prior to COVID-19, data primarily flowed in one direction from healthcare providers to public health. Data and information back from public health was limited in both timeliness and content. Now, with CBOs joining healthcare and public health in the mix, data and information must flow in both directions with near real-time feedback loops. Operational and workflow improvements depend on timely, accurate data and the integration of insights from analytics. Community-based organizations may not have financial resources to bring to bear, but the data they collect more than make up the difference. An example of this was in another HEC-led PPP project to increase vaccination rates in Solano County, Northern California (10, 11). At that time, Solano County Public Health was concerned that its migrant farmworker population had an increased burden of COVID-19 related deaths, which mirrored its significantly lower rates of vaccination. Partnership with Microsoft and Amazon allowed streamlining of workflows and transitioned from manual fax and PDR file to automated data collection. This was consistently performed across sites of care, and data exchanged through a uniform and secure, privacy preserving manner including the cleansing, mapping, transformation and routing of data to appropriate endpoints based on the principles of Minimum Necessary and Appropriate Disclosure (13). The Solano County Public Health platform supports a full breadth of services, including but not limited to: scheduling, case management, registration, SDOH surveys and the data management of vaccinations; as well as solving for the challenge of over-reporting or under-reporting of data to and from public health. This technology is able to support pop-up and mobile vaccination clinics for organizations that do not typically have access to Electronic Health Record Systems (EHRs), such as schools, churches, and non-profit organizations. It also ensured that clinical data flowed seamlessly and securely between CBOs, local healthcare organizations, including federally qualified health clinics (FQHCs), public health, and state immunization registries.

### 2.3. Mutual value creation

A clear understanding and alignment of goals, incentives, skills and resources can create mutual value for all stakeholders involved in a PPP model. The value can result in direct economic gains, such as increasing a provider's capacity to deliver healthcare services, or indirect economic gains, such as with cost savings from improved efficiency of care delivery. HEC delivered measurable value for stakeholders across the health care ecosystem in the following ways:

1. **Healthcare provider.** Reporting requirements from healthcare providers to public health for both infectious and noninfectious diseases vary considerably across states and local territories. Challenges often exist for healthcare providers to adhere to reporting regulations (14), with frequent under-, over- and delayed reporting instances. Over-reporting, such as when an entire medical chart is shared when only a lab result was requested, is a HIPAA privacy violation. The use of real time bidirectional flow of data during the HEC-led PPP initiatives enabled more precise and timely sharing of data between providers and public health in a privacy preserving manner.
2. **Public Health.** Timely collection of public health data from healthcare providers allowed for local, real-time, situational analysis on emerging risk areas and outbreaks during the COVID-19 pandemic. HEC also partnered with ESRI GIS mapping technology (15), to map real time information on COVID-19 and other communicable disease burdens through biosurveillance inputs across various geographies. This allowed public health agencies to plan their prevention and intervention efforts towards the communities that needed it most.
3. **Payer.** Increased vaccination rates, more disease prevention screenings, and improved coordination of care across hard to reach populations increased payer satisfaction ratings and Healthcare Effectiveness Data and Information Set (HEDIS) scores, is a tool used by the majority of U.S. health plans to measure performance on important dimensions of care and service. HEC's community-centric, collaborative approach of convening motivated organizations and providing a community-level environment for collaboration also improved the ability of managed care organizations (MCOs) to identify and enroll people in Medicaid and other state programs.

### 2.4. Data and AI to solve complex problems

As more data and information become available the potential promise of Analytics and AI can be realized. However, it is critical that compliance, privacy, and trust be maintained while also taking steps to mitigate the impact of data, political, and other bias. Confidential computing and the capability to create and share insights without having to share the actual data need to be available to all. New semi-automated, flexible, dynamic, and auditable agreements need to be in place to be able to adapt to real-world changes. AI analysis of the data can be useful for drawing insights on complex problems, such as mapping patterns of transmission for viral and respiratory pathogens, predicting risk factors for subsequent illnesses and predicting future hospitalizations in a certain geographical area. Furthermore, incorporating geographic information into an organization's dashboard, enables spatial planning for targeting public health prevention and intervention measures. For example, HEC worked with Microsoft and Amazon to leverage cloud technologies to enable public health information discoveries including the identification of gaps in reporting, the need to improve mapping of race and ethnicity to avoid errors in reporting and bias in analytics. Vaccination breakthrough analysis leveraged existing data relationships between testing, screening, vaccination, case and other public health data in near real-time while preserving privacy through patient-linked, de-identified data.

## 3. Conclusion

Health equity is a complex, multi-sectoral issue which requires participation by multiple stakeholders to address. Using examples from the Health Equity Consortium (HEC), we describe how technology can be used to facilitate collaborations between public and private entities to address health misinformation, reduce vaccine hesitancy, and increase access to social and primary care services across various underserved communities during the COVID-19 pandemic. We call out four enablers of effective collaboration within the HEC-led PPP model, including: 1. Establishing trust in the population to be served 2. Enabling bidirectional flow of data and information 3. Mutual value creation and 4. Applying analytics and AI to help solve complex problems. The PPP process has promise in that it connects stakeholders with each other and those most in need. Limitations of this HEC-led PPP model to address health inequity is its demonstration only during the period of the COVID-19 pandemic. The timing may have been unique supportive of an effective collaboration between private and public entities, in that all stakeholders and the public were highly engaged in a global public health emergency with a strong desire to “return to normal”. Continued evaluation and improvements to the HEC-led PPP model are likely needed to sustain the model post pandemic. This may include collaborative efforts with federal agencies such as the US Department of Health and Human Services (HHS) (16) and Centers of Disease Control (CDC) (2), both of whom have a long-standing history of engagements with the private sector, to help scale this technology driven PPP model across the U.S. to prove its value in addressing health inequities.

## Data Availability

The original contributions presented in the study are included in the article, further inquiries can be directed to the corresponding author/s.
